# Prader-Willi syndrome: a review of clinical, genetic, and endocrine findings

**DOI:** 10.1007/s40618-015-0312-9

**Published:** 2015-06-11

**Authors:** M. A. Angulo, M. G. Butler, M. E. Cataletto

**Affiliations:** Department of Pediatrics, Winthrop University Hospital, 101 Mineola Blvd, 2nd Floor, Mineola, NY 11501 USA; Department of Psychiatry and Behavioral Sciences and Pediatrics, University of Kansas Medical Center, 3901 Rainbow Blvd, MS 4015, Kansas City, KS 66160 USA; Department of Pediatrics, Winthrop University Hospital, 120 Mineola Blvd, Suite210, Mineola, NY 11501 USA

**Keywords:** Prader-Willi syndrome, Obesity, Chromosome 15 abnormalities, Genomic imprinting, Endocrine disturbances, Short stature, Hypogonadism

## Abstract

**Introduction:**

Prader-Willi syndrome (PWS) is a multisystemic complex genetic disorder caused by lack of expression of genes on the paternally inherited chromosome 15q11.2-q13 region. There are three main genetic subtypes in PWS: paternal 15q11-q13 deletion (65–75 % of cases), maternal uniparental disomy 15 (20–30 % of cases), and imprinting defect (1–3 %). DNA methylation analysis is the only technique that will diagnose PWS in all three molecular genetic classes and differentiate PWS from Angelman syndrome. Clinical manifestations change with age with hypotonia and a poor suck resulting in failure to thrive during infancy. As the individual ages, other features such as short stature, food seeking with excessive weight gain, developmental delay, cognitive disability and behavioral problems become evident. The phenotype is likely due to hypothalamic dysfunction, which is responsible for hyperphagia, temperature instability, high pain threshold, hypersomnia and multiple endocrine abnormalities including growth hormone and thyroid-stimulating hormone deficiencies, hypogonadism and central adrenal insufficiency. Obesity and its complications are the major causes of morbidity and mortality in PWS.

**Methods:**

An extensive review of the literature was performed and interpreted within the context of clinical practice and frequently asked questions from referring physicians and families to include the current status of the cause and diagnosis of the clinical, genetics and endocrine findings in PWS.

**Conclusions:**

Updated information regarding the early diagnosis and management of individuals with Prader-Willi syndrome is important for all physicians and will be helpful in anticipating and managing or modifying complications associated with this rare obesity-related disorder.

## Introduction

Prader-Willi syndrome (PWS) was first described by Prader et al. in 1956 [1] and now recognized as a genomic imprinting disorder whereby differential expression of genes depending on the parent of origin contributes to the imprinting process. Errors in genomic imprinting, which occurs during both male and female gametogenesis are causative for PWS and include the loss of expression of paternal genes, which are normally active and located in the chromosome 15q11-q13 region [2–6]. Conversely, a loss of expression of the preferentially maternally expressed UBE3A gene in this region leads to Angelman syndrome (AS), an entirely different clinical disorder [7, 8]. About two-thirds of individuals with PWS have a de novo paternally inherited deletion of the chromosome 15q11-q13 region. The remaining individuals have maternal disomy 15 (both chromosome 15 s received from the mother with no paternal chromosome 15 present) in about 25 % [9] of cases or have defects in the genomic imprinting center due to microdeletions or epimutations found in fewer than 3 % of cases [2, 4, 10, 11]. On very rare occasions, chromosomal translocations or rearrangements of the 15q11-q13 region are reported [2, 12–17].

With an estimated prevalence of 1/10,000–1/30,000, PWS is the most common syndromal cause of life-threatening obesity and the first recognized disorder related to genomic imprinting in humans [9, 18]. Affected infants uniformly have significant hypotonia, feeding difficulties, and failure to thrive (FTT), followed in later infancy or early childhood by excessive appetite with gradual development of obesity, short stature and/or decreased growth velocity, intellectual disabilities (average IQ of 65), and behavioral problems (e.g., temper tantrums, outburst, and skin picking) [3, 13]. Hypothalamic dysfunction has been implicated in many manifestations of this syndrome including hyperphagia, temperature instability, high pain threshold, sleep-disordered breathing, and multiple endocrine abnormalities [3, 5, 6].

This review summarizes clinical manifestations, genetics and genetic testing, sleep-disordered breathing, and screening with management of endocrine abnormalities associated with PWS.

## Clinical manifestations and characteristic features of PWS

**Fig. 1:**
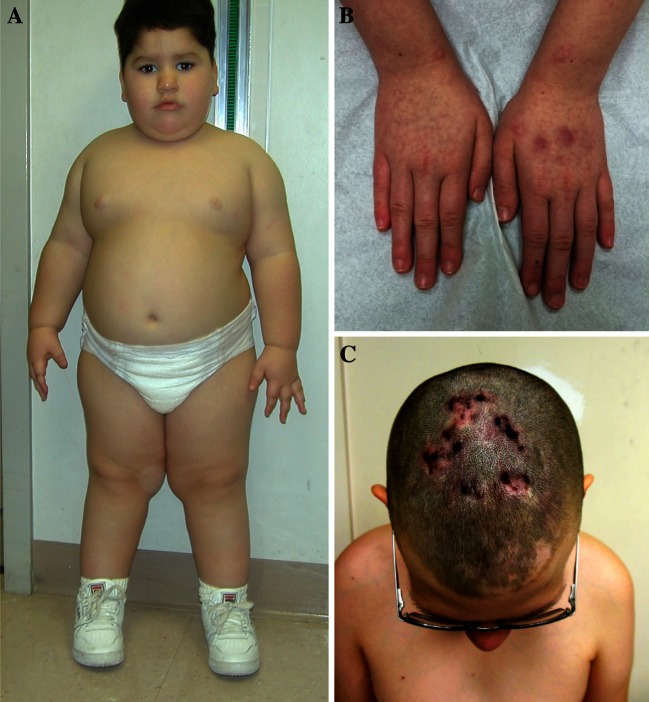
**a** Obesity, almond shape eyes, down-turned mouth and straight borders of inner legs. **b** Straight borders of ulnar side of hands and scares from skin picking. **c** Active and healing skin lesions on scalp

Severe hypotonia is consistently observed at birth and during the neonatal period [3]; therefore, PWS should be considered in all cases of unexplained neonatal hypotonia. Other features noted during the neonatal period include lethargy, feeding difficulties, thick saliva, and increased head/chest circumference ratio, small genitalia in both males and females with frequent cryptorchidism in males. In older untreated children with obesity, developmental delay, short stature and/or decreased growth velocity, and dysmorphic features are found including a narrow bifrontal diameter, almond-shaped palpebral fissures, a thin upper lip with a down-turned mouth, small hands and feet, straight borders of ulnar side of hands and of inner legs (Fig. 1) [3]. Clinical diagnostic criteria were established by consensus in 1993 [19]. Subsequently, definitive molecular genetic testing became available for laboratory diagnosis of PWS. These clinical criteria were later modified to help define people for whom further diagnostic testing is indicated and at different ages (Table 1) [20]. DNA methylation analysis is the most efficient way to confirm the diagnosis if PWS is suspected clinically but will not identify the genetic subtype [3, 21, 22].

**Table 1:** Suggested new criteria to prompt DNA testing for Prader-Willi syndrome (PWS)

Age at assessment	Features to prompt DNA testing for PWS
Birth to 2 years	1. Severe hypotonia and poor suck
2–6 years	1. Hypotonia with history of poor suck
	2. Global developmental delay
	3. Short stature and/or decreased growth velocity
	4. Hypogenitalism/hypogonadism
6–12 years	1. History of hypotonia with poor suck
	2. Global developmental delay
	3. Excessive eating with central obesity, if uncontrolled
	4. Hypogenitalism/hypogonadism
13 years through adulthood	1. Cognitive impairment; usually mild intellectual disability
	2. Excessive eating (hyperphagia; obsession with food) with central obesity, if uncontrolled
	3. Hypogonadism and/or typical behavior problems (including temper tantrums and obsessive-compulsive features)
	4. Short stature; small hands and feet

Adapted from Gunay-Aygun et al. [20]

Classically, two nutritional developmental phases have been described in PWS: Phase 1, in which the individual exhibits poor feeding and hypotonia, often with FTT; and Phase 2, which is characterized by “hyperphagia leading to obesity” [3, 6, 13] but recently a total of seven different nutritional phases, with five main phases and sub-phases in Phases 1 and 2 have been identified [23]. Increase in appetite is seen in Phase 2b at age 4.5–8 years, whereas the classical hyperphagia becomes evident during Phase 3 (Table 2).

**Table 2:** Clinical characteristics of the nutritional phases seen in Prader-Willi syndrome

Phase 0	Decreased fetal movements and lower birth weight than sibs
Phase 1a	Hypotonia with difficulty feeding (0–9 months)
	Needs assistance with feeding either through feeding tubes [nasal/oral gastric tube or gastrostomy tube] or orally with special, widened nipples
	Decreased appetite
Phase 1b	No difficulty feeding and normal growth (9–25 months)
Phase 2a	Weight increasing without appetite increase (2.1–4.5 years)
	Will become obese if given the recommended daily allowance [RDA] for calories
	Typically needs to be restricted to 60–80 % of RDA to prevent obesity
Phase 2b	Weight and appetite are increased (4.5–8 years)
	Will become obese if allowed to eat what they want
Phase 3	Hyperphagic, rarely feels full (8 years-adulthood)
	Constantly thinking about food with temper tantrums related to food
Phase 4	Appetite is no longer insatiable (adulthood)
	Improvement in control of appetite and temper tantrums
	Most adults have not gone into this phase and maybe some (most?) never will

Adapted from Miller et al. [23]

The last two decades have seen significant increases in the understanding of mechanisms controlling appetitive behavior, body composition, and energy expenditure. Many regions throughout the central nervous system play critical roles in these processes but the hypothalamus, in particular, receives and orchestrates a variety of signals to bring about coordinated changes in energy balance. Ghrelin, a 28 amino acid peptide produced in the stomach, is the only peripheral hormone to transmit satiety signal. Plasma ghrelin level in obese PWS individuals is higher than any other form of obesity and considered as one of the contributing factors for their obesity [24, 25]. A somatostatin analog infusion in 4 adults [26] and long–acting octreotide infusion in 8 adolescents with PWS effectively suppressed ghrelin elevation before meals but not the appetite [27]. Circulating ghrelin levels are elevated in young children with PWS long before the onset of hyperphagia, especially during the early phase of poor appetite and feeding [28]. Based on these studies, it seems unlikely that high ghrelin levels alone are directly responsible for the switch to the hyperphagic nutritional phases in PWS.

Diabetes mellitus type 2 (T2 DM), a metabolic disorder characterized by hyperglycemia in the context of insulin resistance has been reported in 25 % of adult PWS population [29]. Those individuals with T2 DM had a higher past maximum body weight and a greater likelihood of positive family history. Fasting insulin concentrations and homeostasis model assessment insulin resistance index however, are lower in PWS children than in obese control (*P* < 0.05) and similar to lean control subjects [30]. Not surprisingly, a study of 74 children with PWS at a median age of 10.2 years showed that none had T2 DM and only 4 % had impaired oral glucose tolerance by OGTT [31]. T2 DM should be managed accordingly with special attention to those children on GH treatment with higher risk for insulin resistance. Periodic fasting serum glucose and insulin levels are recommended before and after initiation of GH treatment.

## Genetics

Errors in genomic imprinting are the cause of PWS. It is considered as a phenomenon of epigenetics and modified dependent on the parental sex contributing the genes where epigenetic changes can control expression or gene activity without changing the DNA structure or base pair sequence [32]. The epigenetic process is reversible and occurs during both male and female gametogenesis. The ‘on or off’ activity of gene expression or regulation is usually through DNA methylation at specific bases (e.g., cytosine). Nearly 150 genes in humans are thought to be imprinted and contain CpG-rich differentially methylated DNA regions that correlate with gene allele activity [18]; several located on chromosome 15.

**Fig. 2:**
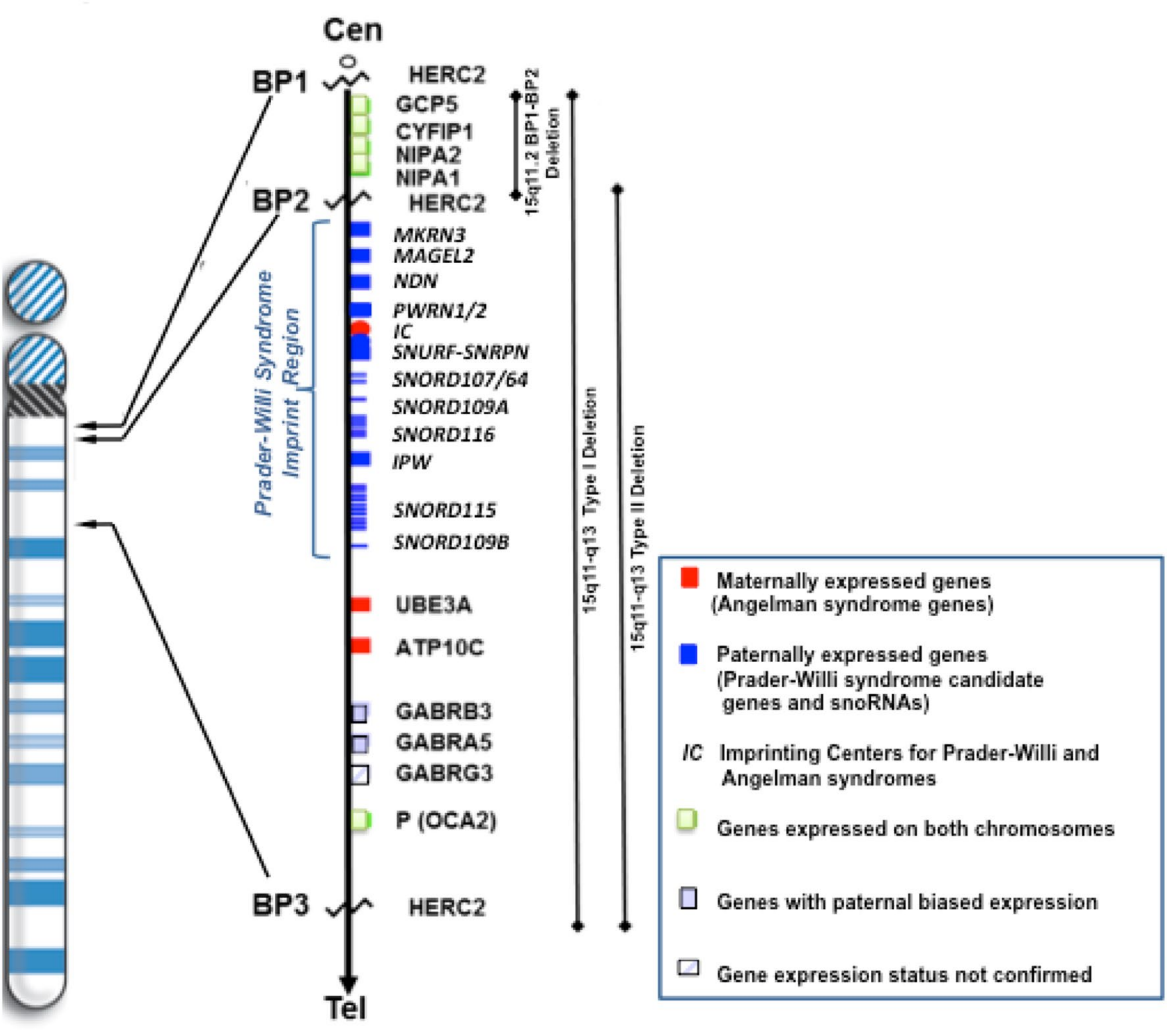
High resolution chromosome 15 ideogram and locations of breakpoints BP1 and BP2 [at 15q11.2 band] and BP3 [at 15q13.1 band] are shown with position of the four non-imprinted genes between breakpoints BP1 and BP2 and those imprinted and non-imprinted genes between breakpoints BP2 and BP3. Three recognized deletion subtypes and their locations in the 15q11-q13 region (i.e., 15q11.2 BP1-BP2; typical 15q11-q13 type I; typical 15q11-q13 type II) are represented

The genes and transcripts located in the 15q11-q13 region can be grouped into four areas delineated by three common deletion breakpoints (Fig. 2): (1) A group of non-imprinted genes, *GCP5, CYFIP1,NIPA1*, and *NIPA2*, are located between proximal 15q11-q13 breakpoints BP1 and BP2 and other genes between 15q11-q13 breakpoints BP2 and BP3 (e.g., *P* gene) are also expressed equally from the paternal and maternal alleles; (2) imprinted genes (paternal expression only) include *MKRN3, MAGEL2, NDN*, and the bicistronic *SNURF*-*SNRPN* gene; (3) the preferentially maternally expressed genes *UBE3A* and *ATP10A* (with disturbances in *UBE3A* causing Angelman syndrome); and (4) non-imprinted genes show evidence of paternally biased expression (e.g., *GABRB3*) [2, 33, 34]. The bicistronic *SNURF*–*SNRPN* gene in the 15q11-q13 region is involved in mRNA splicing in the brain encoded by exons 4 to 10. Exons 1 through 3 of this complex gene encode a separate protein, which is involved in the genomic imprinting process [2]. Multiple copies of the so-called C/D box small nucleolar RNAs (snoRNAs) or SNORDs (*SNORD64, SNORD107, SNORD109A, SNORD115,* and *SNORD116*) are also located in this region and key in the development of PWS with involvement in RNA processing. SNORDs are encoded by a large extended transcript from the complex *SNURF*-*SNRPN* gene locus and not translated into protein [35–38].

Other protein coding genes in the 15q11-q13 region include *MKRN3, MAGEL2,* and *NDN* which are imprinted and paternally expressed. They are located proximally to the imprinting center (IC) located at the *SNRPN*-*SNURF* gene complex locus and involved in neural development and brain function [2, 4, 5]. The *MAGEL2* gene encodes a protein found in the hypothalamus and other brain areas and recently have been reported to be involved in autism spectrum disorder [39]. It appears to function in circadian rhythm, brain structure development, and human reproduction and infertility. The *MKRN3* gene generates specific proteins (makorins), which are abundantly expressed in the brain and involved with hormone regulation and precocious puberty [40]. The *NDN* gene is thought to play a role in axonal outgrowth and expressed in brain regions possibly involved in regulating respiration rate. Two additional genes (*PWRN1, PWRN2*) are also located close to the *NDN* gene with the *PWRN1* as a possible novel alternative start site for the *SNURF*-*SNRPN* gene complex activity [41]. The *UBE3A* and *ATP10C* genes are imprinted, maternally expressed, and paternally silent. The *UBE3A* gene is involved in Angelman syndrome. Several transcripts in the 15q11-q13 region are read in an anti-sense direction and complementary to DNA sequences of other genes in a reverse direction, including the *UBE3A* anti-sense transcript. Other genes found in the distal non-imprinted area of the 15q11-q13 region include the gamma aminobutyric acid (GABA) receptor subunits (i.e., *GABRB3, GABRG3, GABRA5*), the P locus for oculocutaneous albinism type 2 (*OCA2*) and *HERC2* [2, 4, 5]. The *HERC2* gene encodes an ubiquitin ligase protein, which is expressed in high levels in the fetus and lower expression in the adult brain, testis, ovary, and muscle tissue [10, 42].

Studies have shown that the GABA receptor subunit genes (i.e., *GABRB3, GABRA5*) are expressed unequally between the paternal and maternal alleles thereby indicating altered allelic expression. Loss of the paternal allele for these genes produces lower than the expected 50 % expression which indicates paternal bias with more expression from the paternal allele than the maternal allele using lymphoblasts carrying different chromosome 15 defects (e.g., deletions, uniparental disomy) [33, 34]. GABA is an important neurotransmitter with inhibitory capability at the brain level. Thus, alterations in gene expression may be associated with appetite, visual perception, and memory changes. The P gene is involved with pigmentary status in individuals with PWS or Angelman syndrome having the typical chromosome 15q deletion. This oculocutaneous albinism type 2 gene when disturbed in individuals with PWS or Angelman syndrome having the typical 15q11-q13 deletion will show hypopigmentation [2, 4, 5, 43].

## Genetic subtypes and diagnostic testing for Prader-Willi syndrome

There are three main genetic mechanisms that result in PWS: paternal 15q11-q13 deletion, maternal uniparental disomy (UPD) 15, and imprinting defects (ID).

### Paternal deletion

Two proximal chromosome 15q11-q13 breakpoints (BP1 and BP2) and a distal breakpoint (BP3) appear to predispose to the typical deletions seen in PWS (65–75 % of cases) and Angelman syndrome [2, 4, 5]. The most common typical deletions are of two classes, Type I and Type II (Fig. 2). The Type I deletion is larger and involves the proximal breakpoint BP1 which is nearest to the chromosome 15 centromere while the smaller Type II deletion involves the other proximal breakpoint BP2. The third common breakpoint (BP3) is located distally in this chromosome region and is involved in both typical deletion types [44]. Four genes are located in the genomic area between proximal breakpoints BP1 and BP2 including *GCP5, CYFIP1, NIPA1,* and *NIPA2*. These genes are overly expressed in the brain and when mutated (e.g., *NIPA1*) can lead to spastic paraplegia and brain disturbances [45, 46]. PWS individuals with the smaller Type II deletion have these four genes intact. Individuals without PWS are reported with behavioral and autistic findings when only a deletion is present involving the region between breakpoints BP1 and BP2, the chromosome 15q11.2 BP1-BP2 microdeletion (Burnside-Butler) syndrome [47–49].

PWS individuals with the larger Type I deletion have been reported to be more prone to obsessive compulsion and self-injury (skin picking) in addition to visual processing deficits and lower measures of academic performance than those PWS individuals with the smaller Type II deletion having the four genes intact between proximal breakpoints BP1 and BP2 [50, 51]. Other deletions that are not typical and vary in size involving areas of the 15q11-q13 region are reported in about 5 % of PWS individuals [2, 4, 5, 16]. These individuals are often more atypical in their clinical presentation [15].

The smallest genetic defects in the 15q11-q13 region often include microdeletions of the paternally expressed non-coding snoRNAs such as *SNORD115* and *SNORD116* [15, 35–37]. Studies in mice containing disturbances of *SNORD116* equivalent transcripts exhibit hyperphagia and growth failure, which are common features in PWS. In addition, research supports that *SNORD115* regulates alternative splicing of the human serotonin 5-HT2C receptor providing an altered receptor form leading to excessive eating behavior [38], a cardinal feature seen in PWS.

### Maternal uniparental disomy (UPD) 15

The second most frequent genetic finding in PWS is due to an error in meiosis, most common when two maternal chromosome 15 s are contributed in the egg and fertilized by a normal sperm. This leads to 47 chromosomes in the fetus causing trisomy 15. Trisomy 15 is a relatively common cause of early miscarriages in humans. If a trisomy 15-rescue event occurs, then one of the chromosome 15 s will be lost from the trisomic cell leading to a normal 46 chromosome count with continuation of the pregnancy. If the paternal chromosome 15 is lost then the cell will have two maternal chromosomes 15 s. The fetus will survive with two maternal chromosomes 15 (UPD) with the clinical picture of PWS but normal cytogenetic findings at the time of delivery [52].

Maternal uniparental disomy (UPD) 15 in PWS can be of three types: (1) heterodisomy, as a result of non-disjunction of homologous chromosome 15 s in meiosis I, thus the baby inherits each of the mother’s two chromosome 15 s; (2) isodisomy, as a result of non-disjunction in meiosis II with two identical chromosome 15 s inherited from the mother; and (3) segmental form occurs when regions of chromosome 15 have identical genetic information as a result of crossing-over events and non-disjunction in meiosis I or possibly by a somatic chromosome recombination in early pregnancy. The type of disomic event may impact on the pregnancy and clinical outcome of the fetus. Most PWS subjects with maternal disomy 15 have the heterodisomic form [4, 5, 53].

Extra attention however, should be given to PWS individuals with maternal disomy 15 if the isodisomic or segmental type is present by examining for additional genetic disorders due to mutant recessive genes carried on the maternal chromosome 15q. Special genetic testings such as high-resolution DNA microarrays with SNP probes are recommended to assist in identifying the disomic status. As in other non-disjunction cases, the risk of maternal disomy 15 increases with maternal age. Those PWS individuals with maternal disomy 15 often have delayed diagnosis, higher verbal IQ scores with greater attention, and factual knowledge and better social reasoning skills than those with the typical Type I or Type II deletions involving the 15q11-q13 region but are more prone to increased episodes of psychosis and autistic behaviors [45, 50, 51].

### Imprinting defects (ID)

Most individuals with PWS are due to sporadic causes but in some families the defective error is from an epimutation or incomplete processing of the imprint in germ cell meiosis from the father or from a microdeletion of the DNA imprinting center (1–3 %). The microdeletion defect has been reported in about 15 % of individuals with PWS due to ID [11], although more recent studies indicate a possible higher rate for microdeletions in the imprinting center [54]. This microdeletion can be contributed by the paternal grandmother to the father and lead to birth of another child with PWS. The risk in this situation is 50 %.

### Diagnostic genetic testing

DNA methylation provides a powerful tool to assess paternal-only, maternal-only, or biparental (normal) inheritance. Normal individuals have both a methylated and an unmethylated allele, whereas individuals with PWS have only the maternally methylated allele, therefore the most efficient analysis to diagnose PWS. The most widely used DNA methylation analysis only targets the 5′ CpG island of the *SNRPN* locus and will correctly diagnose PWS in more than 99 % cases but can not distinguish between a deletion, UPD or ID [3]. A more recent generation of DNA methylation assay, “methylation-specific multiplex-ligation probe amplification” (MS-MLPA) is more informative. MS-MLPA will determine the methylation status by using 5 to 6 methylation probes in the *SNRPN* locus and other imprinted genes close by to confirm the diagnosis of PWS as well as about 30 probes within the 15q11-q13 region that are used with reference (control) probes outside of the region and on other chromosomes to determine the copy number status [21, 22]. This assay will identify the typical deletion, which is seen in the majority of individuals with PWS as well as the methylation status. If the deletion is not seen with MS-MLPA testing and the PWS methylation pattern is present, then high-resolution microarrays including SNP probes should be used to help identify an imprinting defect or maternal disomy 15 status. In some families more testing will be needed including genotyping of chromosome 15 DNA markers using parental DNA.

## Sleep disruption and sleep-disordered breathing

Sleep disruption and sleep-disordered breathing have been linked to significant deficits in neurocognitive function, including poor focus, excessive daytime sleepiness, and irritability in both the general population [55] and in individuals with PWS [56]. Initially prompted by the daytime feature of hypersomnolence, many individuals with PWS were identified with polysomnographic features of sleep-disordered breathing, including obstructive, central, and mixed sleep apnea syndromes. Factors including developmental brain abnormalities, craniofacial dysmorphia, hypotonia, obesity, and chest wall deformities have been cited as factors that can contribute to both the presence and severity of sleep-disordered breathing in PWS [57]. Following their metanalysis of the literature on sleep-disordered breathing in PWS, Sedky et al. [57] concluded that while obstructive sleep apneas (OSA) were closely related to obesity in non-PWS children, it was unclear whether body mass index (BMI) played a significant role in increasing OSA risk in PWS children. Central apneas were more common in infants studied with PWS [58]. Symptomatic narcolepsy, with or without cataplexy has also been reported in up to 35.7 % of children with PWS [57].

Hypocretin containing neurons, located in the hypothalamus are thought to play an important role in maintaining wakefulness as well as influencing eating behavior. Congenital dysfunction/developmental failure of the hypocretin system has also been proposed as a potential etiology in this population [59–61]. In genome wide expression studies, Bittel et al. [62] reported elevated expression patterns of the hypocretin (*HCRT*) gene in males with PWS, although an early study by Fronczek et al. [63] did not show a significant difference in the total number of hypocretin-containing neurons among PWS patients and age-matched controls, either in adults or infants.

Excessive daytime sleepiness (EDS) is a very common feature in PWS, occurring in 70–100 % of adults with PWS. Most recently, Mass et al. [64] utilized actigraphic scatter-plots to explore the temporal distribution of EDS with severe disruptive behavior and to identify situations where sleepiness was most likely to occur. Lack of structured activities, particularly in the afternoons and evenings were associated with higher rates of EDS.

GH is now commonly used in the management of PWS. The effect of GH during wakefulness has demonstrated improvements in respiratory mechanics and ventilatory responsiveness [65]. More recently, Katz-Solomon et al. [66] performed studies to assess cardiorespiratory control during sleep following the initiation of GH. They demonstrated improved oxygenation and cardiovascular function at 6 months after the initiation of GH in 16 individuals between ages 2–32 months. However, their study also showed that the ventilatory response to 4 % CO_2_ and 100 % O_2_ was essentially unchanged during quiet sleep suggesting that the previous changes were unrelated to an improvement in chemoreflex-mediated autonomic drive [66].

Longitudinal follow-up studies performed after 2 years of GH therapy reported by Al-Saleh et al. [67] in 15 children between 0.8 and 15.4 years (median 3.7 years) showed a median obstructive apnea/hypopnea index (OAHI) of 0.8 (0.3–6.1) and central apnea index (CAI) of 0.9 (0.2–2.3). GH was discontinued in two of these children due to the development of severe OSA identified by polysomnography at 6 weeks post-initiation of GH therapy. However, at 2 years post-treatment no significant changes in sleep-disordered breathing were identified in these children, suggesting a period of increased vulnerability in the first few weeks after the initiation of treatment [67].

Berini et al. [68] evaluated adenotonsillar size in 50 children with PWS before the initiation of GH treatment at 6 weeks, at 6 months, at 12 months, and then yearly up to 4 years. Three children developed severe OSA requiring discontinuation of growth hormone therapy. This group found a direct correlation of OAHI with adenoid size but neither with tonsillar size nor with plasma IGF-1 levels [68]. Thus, it is appropriate to screen all individuals with PWS for sleep-disordered breathing. While screening questionnaires and physical examinations may be helpful, neither has shown good specificity or sensitivity [69]. Opinions vary as to the timing and frequency of sleep evaluations but most agree that it is appropriate to study children before the initiation of GH and then periodically, especially with significant changes in weight, prior to and following adeno-tonsillectomy and before spinal or craniofacial surgery.

Whitman and Myers [70] also recommended a repeat polysomnogram at 3–6 months after the initiation of GH in patients with PWS previously diagnosed and treated for OSA as well as regular screening with the Chervin sleep questionnaire (questions 1–6), examination of the oropharynx for tonsillar hypertrophy and monitoring of IGF-1 levels during the entirety of GH therapy. Multiple sleep latency testing (MSLT) is also useful in assessing pathologic sleepiness and is done following an overnight polysomnogram.

In children, adeno-tonsillectomy is the preferred treatment for obstructive sleep apnea. High-risk children including those with PWS and especially those with severe apnea may have residual apneic events and require post-operative evaluation to assess if additional therapies, such as Continuous Positive Airway Pressure (CPAP) are needed [71, 72]. Velopharyngeal dysfunction with hypernasality requiring additional surgical intervention has also been reported following adeno-tonsillectomy in children with PWS [73]. In adults with PWS, CPAP is recommended as the initial therapy due to OSA [74].

## Growth hormone (GH) deficiency

Short stature is a main characteristic of individuals with PWS. Children with PWS fail to show the growth acceleration seen in puberty and the mean final height without treatment is 148 cm in girls and 155 in boys [75, 76]. Growth charts for non-GH-treated infants and children with PWS have been developed [77, 78]. Non-PWS obese children have decreased GH secretion while maintaining normal serum insulin-like growth factor-1 (IGF-1) levels and normal height. Children with PWS have short stature with low serum GH and IGF-1 levels, therefore true GHD [76, 79–81] is considered.

Children with PWS treated with GH through childhood are able to achieve normal adult height [75, 82]. Beneficial effects on body weight, body composition, and exercise capacity found in these studies prompted studies looking at the effects of GH unrelated to height. In one study, 60 prepubertal children ages 3.13–7.16 years had normal height and positive long-term effects on maintaining body composition after 8 years of GH treatment without any adverse effect on glucose homeostasis, serum lipids, blood pressure, and bone maturation [83].

Anabolic effects of GH including increase in lean body mass, motor strength, and decrease in fat mass were reported in 21 children when started at 13 ± 6 months of age for a period of 6 years and compared with 27 untreated children of similar age [80]. A recent study reported low BMI in 61.5 % (33.8 % osteoporosis and 27.7 % osteopenia) of 101 patients with a mean age of 5.4 years (range 3–17 years.) and significant improvement after GH treatment for a mean period of 54 months (range 6–144 months) [84].

The beneficial effect of GH therapy in childhood into adulthood is unclear after GH is discontinued. A report of GH treatment discontinuation after 12 and 24 months in 14 individuals with PWS revealed an increase in BMI-SDS (*P* = 0.008 and *P* = 0.003) and visceral fat (*P* = 0.062 and *P* = 0.125), respectively [85]. In one study, however, improved mean BMI (32.4 vs. 41.2), body composition, lower mean hemoglobin A1c, lower mean insulin resistance, and less hypertension were reported in 20 adults (mean age 25.4 years) with PWS at 7.0 ± 4.4 years after discontinuing treatment initiated at age 11.8 ± 2.7 years when compared with 40 untreated PWS adult group [86]. Butler et al. [87] studied 11 adults with PWS (average age = 32 years) over a 2-year period with GH treatment during the first year only. Total lean muscle mass and moderate-vigorous physical activity and plasma IGF-1 and high density lipid (HDL) levels were significantly increased while on GH, while percent body fat decreased during the 12 months of GH treatment. IGF-1 and HDL levels returned to near baseline and body fat increased after GH treatment during the second year. In addition to improved body composition, increase in muscle strength and exercise tolerance has been reported after 12 and 24 months of GH treatment in 15 obese adults with PWS [88]. Cardiovascular features in obese PWS adults including smaller left ventricular size and lower systolic function are similar to those reported in adult GHD [89]. In one study, left ventricular mass increased significantly after 1 and 2 years of GH treatment (0.40 ± 0.11 to 0.97 ± 0.17 mg/days) in 9 adult PWS individuals without evident abnormalities of systolic and diastolic function [90]. These data indicate that beneficial effects of GH are still present even after the epiphyses are fused and long-term GH treatment, in addition to strict diet and exercise program may be necessary to maintain good body composition.

Beneficial cognitive effects have been reported in children [91–93] and adults [94] with PWS during GH treatment. Recent study in 19 children with PWS at median age 6.3 years, showed no changes in cognition and behavior over one or two years of GH treatment [95]. These children however, had marked deterioration in behavior at 6 months after abrupt GH discontinuation. These studies demonstrated that GH maintenance therapy may prevent or slow down the progression of behavioral problems in PWS individuals.

Recombinant human growth hormone (hGH) was FDA approved in the United States in 2000 for the indication of short stature and growth failure due to PWS. In Europe, growth retardation is not required whereas improvement of body composition is included in the approved indication of GH therapy in PWS [96]. Recommended starting dose in children is 0.18–0.3 mg/kg/week given as daily subcutaneous injections with careful monitoring of clinical status, bone age, and serum IGF-1 levels at regular intervals. Potential concerns related to excessively high IGF-1 levels include lymphoid hyperplasia leading to OSA and a theoretical increase in malignancy risk. In one study, IGF-1 and IGF-binding protein-3 (IGFBP3) levels were evaluated over a 2-year period in a group of 33 children with PWS treated with GH. These subjects were compared to 591 subjects treated for GH deficiency. The PWS group had significantly higher IGF-1 levels despite lower doses of GH. However, there was no significant difference in IGF-1 to IGFBP3 molar ratios between the groups, suggesting that bioavailable IGF-1, and therefore risk for adverse effects, may be similar in both groups [97]. In view of existing information, GH dose based on ideal rather than actual body weight and monitoring serum IGF-1 levels between 0 and +2 SDS and/or IGF-1/IGFBP3 molar ratio without exceeding GH pretreatment should be considered to prevent abnormal serum IGF-1 elevation, insulin resistance, and occasional acromegaloid features seen in children with PWS at standard recommended GH dose.

The prevalence of scoliosis in PWS is high (30–80 %) [98, 99]. Scoliosis is a major concern for patients with PWS treated with GH. Prevalence, onset, and progression of scoliosis are not affected by the genotype or by growth hormone treatment [75, 100–103]. Therefore, scoliosis is no longer a contraindication for GH treatment in children with PWS. However, due to the high prevalence of scoliosis and the potentially associated morbidities in patients with PWS, regular physical examinations and periodic radiographic evaluations of the spine are recommended.

Significant attention has been given to the association of GH therapy and sudden death in PWS. In a review of 64 cases of death in children from a few days to 19 years of age, 28 subjects (44 %) received GH treatment [104]. Respiratory disorders were the most common cause of death among treated and untreated patients. In this review, most of the deaths in GH-treated children (75 %) occurred during the first 9 months after the initiation of GH treatment. High mortality rate and increased risk of sudden infant death have been reported in children with PWS independent of GH therapy and without evidence of an association between death and GH treatment [105, 106]. In view of these data, the initiation of GH is recommended at the lower dose, 0.18 mg/kg/week in infants with PWS.

Benefits have been reported with increased lean body mass and decreased body fat mass after 6–12 months GH treatment in adults [107–110]; however, GH is not FDA approved for adult PWS individuals unless confirmed by standard adult GH stimulation testing. The prevalence of GH deficiency in adults with PWS ranges from 15 to 95 %, depending on the agents used for stimulation testing and the threshold GH level used to define deficiency [107, 108]. A normal IGF-1 level does not exclude the diagnosis of GHD and provocative testing is mandatory to make the diagnosis of adult GH deficiency (AGHD). The Endocrine Society (ES) recommends that the insulin tolerance test (ITT) and the GHRH- arginine test are sufficiently sensitive and specific to establish the diagnosis of AGHD. Glucagon stimulation test can also be used when GHRH is not available and performance of an ITT is either contraindicated or not practical in a given patient [111]. It is recommended that GH dosing regimens be individualized rather than weight-based and to start with low doses (0.1–0.2 mg) then titrated according to clinical response, side effects, and IGF-1 levels.

## Hypogonadism

The term “congenital hypogonadism” refers to complete or partial pubertal failure due to insufficient secretion of the pituitary gonadotropins LH and FSH and gonadal sex steroids. Hypogonadism represents a common clinical feature in PWS. Clitoral and labia minora hypoplasia in females and micropenis with hypoplastic scrotal sac in males are evident at birth. Unilateral or bilateral cryptorchidism is present in 80–90 % of males [5]. Similar to many other manifestations of PWS, hypogonadism has been classically thought to be hypothalamic in etiology. However, recent evidence has emerged supporting primary gonadal failure as a significant contributor to male hypogonadism [112–114]. Other studies have also shown a combined picture of hypogonadotropic hypogonadism with relatively low LH levels, and primary hypogonadism with low inhibin B and relatively high FSH levels [114, 115].

The transient increase in gonadotropins and gonadal hormonal levels occurs during the first months of life, and “minipuberty” is normal in infants with PWS [116, 117]. Gonadotropins and testosterone play important roles in testicular descend. If hypogonadotropic hypogonadism is present in boys, decreased LH, FSH, testosterone, and inhibin B levels are found in addition to a micropenis (stretched penis <2.5 cm) and cryptorchidism. These findings suggest that additional factors may be responsible for the high incidence of cryptorchidism in infants with PWS.

Gonadal function has also been evaluated longitudinally in 61 girls with PWS. LH levels were found to be relatively low for the low estradiol levels observed, and FSH levels were normal. Although the girls had a normal onset of puberty, the progression was delayed in comparison to the normal population [118]. The pattern of gonadal dysfunction in PWS females (primary and combination of a primary gonadal defect and hypothalamic dysfunction) seems to be similar to those observed in boys, male adolescents, and adults with PWS [119, 120]. Four females with PWS diagnosed as children (3 with deletions and one with UPD) with onset of menarche in their 20 s became pregnant and delivered children by caesarian section. Two of these mothers had normal children and the two PWS mothers with 15q11-q13 deletions delivered a male and a female with Angelman syndrome also with the deletion [121, 122].

Due to high prevalence of premature adrenarche in PWS [123–125], commonly associated with pubic or axillar hair, careful assessment, preferably using a Prader orchidometer, is necessary to demonstrate further testicular enlargement as a first sign of puberty in males (testicular volume greater than 4 ml indicates onset of puberty) and breast development Tanner stage II as the first sign of puberty in females with PWS. During adolescence, the laboratory diagnosis of hypogonadotropic hypogonadism is relatively simple with the identification of very low circulating total testosterone and low to low-normal gonadotropin and inhibin B levels. This hormone profile rules out a primary testicular disorder in which testosterone and inhibin B levels are low, whereas FSH and LH are elevated. Serum FSH, LH, estrogen, and inhibin B profiles are recommended in females with PWS before considering sex hormone replacement therapy (SHRT).

The beneficial effect of sex steroids on the muscle mass and bone health in an individual is well known. Previous studies have suggested that sex steroid deficiency contributes to low bone density in adults with PWS [126, 127]. However, no consensus statement exists as to the most appropriate regimen of sex hormone treatment in PWS. The choice of a particular hormone replacement therapy protocol will depend on the age at diagnosis and local practices. In a recent study, the administration of human chorionic gonadotropin (hCG) 250–500 IU intramuscular biweekly injections for 6 weeks in 16 infants (median age 1.6 years) with PWS with undescended testes resulted in an anatomical lower testis position in most and 23 % had complete scrotal descent. Orchidopexy was required in 76 % of cases in order to ensure a stable position in the scrotum [128]. Furthermore, the American Academy of Pediatrics, Committee on Genetics recommends a therapeutic trial of hCG before considering surgery for undescended testes [129]. The benefits of this modality of treatment include avoidance of general anesthesia, increased scrotal sac size and phallus length to facilitate circumcision/micturition, and thereby improving surgical outcomes for undescended testes.

Testosterone replacement treatment can also lead to improvement in quality of life in males with hypogonadism [130]. In general practice, injectable testosterone is preferred for reasons of convenience and cost. Testosterone enanthate (TE), one of the preparations available on the international market, can be injected once every 2 or 3 weeks in adult males with hypogonadism [131]. It has been thought that TE may increase aggressiveness, but this has not been clearly demonstrated. However, it seems reasonable to start as low as 25 % of the recommended normal adult TE dose (200–250 mg) with gradual increase as tolerated to keep low-normal serum testosterone levels in males while under an endocrinologist’s guidance. Virilization of male patients can also be achieved by percutaneous testosterone administration in gel or patch form. However, these alternatives are expensive and require daily administration, raising problems of adherence and the risk for skin irritation and skin picking behavior in PWS.

Guidelines for hormonal replacement therapy in females with PWS are tailored individually depending on sexual development, hormonal profiles, bone density, and emotional and social needs. Oral estrogen alone or in combinations with progestin is well tolerated. Girls with PWS have normal or near normal secondary sexual characteristics including breast development and menses may occur indicating a lesser degree of hypothalamic-gonadal dysfunction in females and therefore counseling about the risk of pregnancy and discussion of birth control during reproductive age would be advisable.

## Hypothyroidism

Similar to other endocrinopathies in PWS, the etiology of hypothyroidism is thought to be central in origin. Hypothyroidism has been reported in approximately 20–30 % of children with PWS [132]. Yet, in one study of 47 individuals with PWS age 10–44 years, the prevalence of hypothyroidism was 2.1 % and not different than the normal population of 2 % [133]. Based on low total (T4) and free thyroxine (FT4) in the presence of normal thyroid-stimulating hormone (TSH), one study reported a prevalence of 72.2 % of central hypothyroidism in children with PWS less than 2 years of age [134]. A recent study revealed normal newborn screening in 23 neonates with PWS for congenital hypothyroidism. In the same study, TSH response to thyroid-releasing hormone (TRH), T4 and FT4 in 21 children from birth to adolescence showed normal patterns except in one elder child with central hypothyroidism [135]. Based on these studies, the prevalence of hypothyroidism is variable and cannot be clearly established. Thus, levothyroxine treatment should not be routinely prescribed in children with PWS unless confirmed by thyroid function testing. It is recommended that baseline thyroid function testing (FT4 and TSH) be done during the first 3 months of life unless they have had a normal newborn screening and annually thereafter, especially if the patient is receiving GH therapy.

## Adrenal insufficiency

Based on generalized hypothalamic dysfunction, children and adults with PWS are at risk for central adrenal insufficiency (CAI). The first published cross-sectional analysis in 2008 of adrenal insufficiency in children with PWS, reported CAI in 60 % of cases after receiving an overnight single-dose of metyrapone [136]. However, five subsequent studies using different methodologies, including low- and high-dose Synacthen and insulin tolerance testing did not confirm the reported high frequency of CAI [137–141].

Isolated ACTH insufficiency is rare but it may be a component of a multiple hormone deficiency (MPHD). Development of additional pituitary hormone deficiencies in children with acquired MPHD or initially diagnosed with growth hormone deficiency (GHD) is characterized by a sequential order of hormonal loss, usually GH, TSH, LH/FSH, and ACTH [142]. In adults, LH/FSH disturbances have been reported after GH problems, then followed by TSH and with ACTH being the last pituitary hormone deficiency [143]. Although additional inherent and exogenous factors may regulate adrenal androgen production, normal ACTH secretion action is needed for adrenarche [144–146], which is increased in children with PWS. The true prevalence of CAI in PWS therefore remains unclear with no consensus present among endocrinologists as to whether evaluation should be done for CAI and/or glucocorticoid treatment preoperative or during significant stress.

Metyrapone (30 mg/kg orally at midnight) inhibits 11-hydroxylase, the final step in cortisol synthesis, thereby decreasing cortisol secretion and subsequently increasing ACTH secretion. Using metyrapone is a cumbersome test that is rarely performed because of the difficulty in obtaining metyrapone and the risk of precipitating an adrenal crisis. Hence, parents of children with PWS should be counseled about symptoms consistent with adrenal insufficiency and physicians alerted to the risk and treated accordingly should symptoms occur. In summary, PWS is a classic example of a genetic disorder requiring a complex multi-discipline approach to treat ongoing growth, medical, and endocrine disturbances common to all individuals throughout the natural history of this condition.
